# A novel patient-derived cutaneous melanoma cell line reveals key features of metastatic melanoma

**DOI:** 10.3389/fonc.2025.1531013

**Published:** 2025-07-18

**Authors:** Ana Tomás, Lúcia Roque, Inês Francisco, Ana Luísa Silva, Hugo Nunes, Emanuel Gouveia, Elisabete Carvalho, Victor Farricha, Cecília Moura, Joaninha Costa Rosa, Pedro Miguel Garrido, Cristina Albuquerque, Patrícia M. Pereira, M. Guadalupe Cabral, Marta Pojo

**Affiliations:** ^1^ Unidade de Investigação em Patobiologia Molecular, Instituto Português de Oncologia de Lisboa Francisco Gentil E.P.E., Lisboa, Portugal; ^2^ NOVA Medical School, NOVA University of Lisbon, Lisboa, Portugal; ^3^ Serviço de Oncologia Médica, Instituto Português de Oncologia de Lisboa Francisco Gentil E.P.E., Lisboa, Portugal; ^4^ Unidade de Investigação Clínica, Instituto Português de Oncologia de Lisboa Francisco Gentil E.P.E., Lisboa, Portugal; ^5^ Serviço de Cirurgia, Instituto Português de Oncologia de Lisboa Francisco Gentil E.P.E., Lisboa, Portugal; ^6^ Serviço de Dermatologia, Instituto Português de Oncologia de Lisboa Francisco Gentil E.P.E., Lisboa, Portugal; ^7^ Serviço de Anatomia Patológica, Instituto Português de Oncologia de Lisboa Francisco Gentil E.P.E., Lisboa, Portugal

**Keywords:** cutaneous melanoma, primary cell culture, targeted therapy, immunotherapy, phenotypic plasticity

## Abstract

**Introduction:**

Cutaneous melanoma (CM) is an aggressive form of skin cancer, with rising incidence and poor prognosis at advanced stages. While early-stage CM typically carries a favorable prognosis, a small subset of patients relapses and progresses to advanced disease, highlighting the need for a deeper understanding of CM biology. Here, we describe the establishment and characterization of a novel patient-derived primary cell line, MelT79, developed from a metastatic lesion of a patient initially diagnosed with stage IB CM, who unexpectedly progressed to advanced disease.

**Methods:**

MelT79 was characterized by multicolor fluorescence in situ hybridization, high resolution comparative genomic hybridization, and targeted next-generation sequencing. Gene and protein expression were evaluated by RT-qPCR and immunofluorescence, and cell proliferation was assessed using trypan blue exclusion and BrdU incorporation assays. Sensitivity to BRAF inhibition was measured with the Cell Counting Kit-8 viability assay. Gene and protein expression, proliferation, and drug sensitivity were compared to commercially available CM cell lines.

**Results:**

MelT79 exhibits a complex karyotype with significant chromosomal alterations, including deletions affecting key genes such as CDKN2A/B, SPRED1, and B2M, implicated in melanomagenesis and therapy resistance. Additionally, MelT79 harbors both the BRAF V600E mutation and a rare RET S649L mutation, which has not been previously reported in CM. RET S649L was also identified in an earlier metastatic lesion, possibly conferring a selective advantage, and highlighting this mutation as a potential therapeutic target. Phenotypically, MelT79 displays both differentiation and invasive traits, suggesting that its heterogeneity might contribute to progression and therapy resistance. Furthermore, the cell line exhibited moderate sensitivity to BRAF and MEK inhibitors when compared to other commercially available cell lines, reflecting its heterogeneity.

**Conclusion:**

MelT79 represents a valuable model for understanding CM heterogeneity, progression, and resistance mechanisms, offering new avenues for novel therapeutic interventions in CM.

## 1 Introduction

Cutaneous melanoma (CM) arises from the malignant transformation of melanocytes, the skin’s melanin-producing cells (1). While CM accounts for less than 5% of yearly skin cancer cases, it has a higher potential to metastasize than other cancers, contributing significantly to its high mortality rates (2, 3). In 2022, nearly 60 thousand CM-related deaths occurred globally, comprising approximately 60% of all skin cancer-related deaths (3). Moreover, CM’s incidence has been rising rapidly, with over 300,000 new cases diagnosed in 2022 and projections suggesting up to 500,000 new cases by 2040 (3–5).

Ultraviolet radiation exposure is a major risk factor for CM, causing DNA damage that leads to a high mutational burden, distinctive of CM. *BRAF*, *NRAS* and *NF1* mutations are the most common, resulting in the constitutive activation of the MAPK pathway and promoting uncontrolled cell proliferation (1).

Early-stage CM can often be successfully managed with surgical excision alone, with 5-year melanoma-specific survival rates exceeding 97% for low-risk stage I patients (6). Consequently, clinical guidelines do not recommend routine molecular testing for actionable mutations, such as in *BRAF*, at these initial stages (7). However, up to 13% of these low-risk patients experience relapse within 5 years of diagnosis, highlighting the need for improved risk stratification in early CM stages (8, 9).

At advanced stages III-IV, surgery alone is insufficient, and molecular testing is mandatory to better guide therapeutic decisions (7). *BRAF*-mutant CM can be treated with selective BRAF and MEK inhibitors (BRAFi, MEKi), although resistance often develops within a year (1, 7). Immunotherapies, namely immune-checkpoint inhibitors anti-PD-1 and anti-CTLA-4, have shown more lasting responses, but primary and secondary resistance remains a challenge (7, 10, 11). Advanced CM prognosis remains poor, with only 23% of stage IV patients surviving beyond 5 years (12).

To improve outcomes, a deeper understanding of CM biology and identification of new biomarkers and therapeutic targets is essential. Patient-derived primary cell cultures play a key role in this effort, providing insights into how different CM phenotypes drive tumor progression and therapeutic resistance. Expanding the range of available cell lines allows for a more comprehensive representation of CM’s heterogeneity (13). However, developing these cultures poses significant challenges, including fibroblast overgrowth and difficulty in preserving the original tumor’s characteristics *in vitro* (13, 14). Here, we describe the establishment of a new patient-derived CM primary cell line, which we have designated MelT79, along with its molecular and phenotypic characterization.

## 2 Materials and methods

### 2.1 Patient history

A 50-year-old male patient was diagnosed with stage IB cutaneous melanoma (according to the 8th Edition of the American Joint Committee on Cancer (15)) in 2009 after surgical resection of a 2 mm thick lesion on the right thigh. Sentinel node biopsy showed no metastasis, and the patient remained disease-free for 4 years. In 2013, a satellite cutaneous metastasis was excised. In 2014, a nodule on the right inner thigh led to a wide excision, revealing intranodal metastases in 5 lymph nodes. A subsequent right inguinal lymphadenectomy showed no further metastasis in 34 lymph nodes.

In 2015, a cutaneous nodule was excised from the thoracic wall, confirmed to be a melanoma metastasis harboring the BRAF V600E mutation. Additionally, right iliac lymph node metastases were excised, and the patient began treatment with Vemurafenib plus Cobimetinib in 2016. A 2017 PET scan showed right external iliac adenopathy, and the patient remained under surveillance, as he had already undergone surgery in that region.

In 2021, PET scan revealed right adrenal gland metastasis, prompting an adrenalectomy. Later that year, a bleeding cutaneous metastasis was excised from the abdominal wall, but a subsequent PET scan showed disease progression with lymph node, subcutaneous, intramuscular, and perirenal involvement. The patient discontinued targeted therapy and started anti-PD-1 with pembrolizumab.

By April 2022, further metastases in the left thorax and hypochondrium prompted a segmental resection of the colon. As the disease progressed, the patient underwent surgical resection of multiple metastatic foci in the intra-abdominal, retroperitoneal, cervical, gluteal and thoracic regions in early 2023. However, with continued disease progression, pembrolizumab treatment was discontinued in August, and the patient began a new regimen with ipilimumab plus nivolumab (induction treatment), followed by maintenance of nivolumab monotherapy to date.

**Figure 1:**
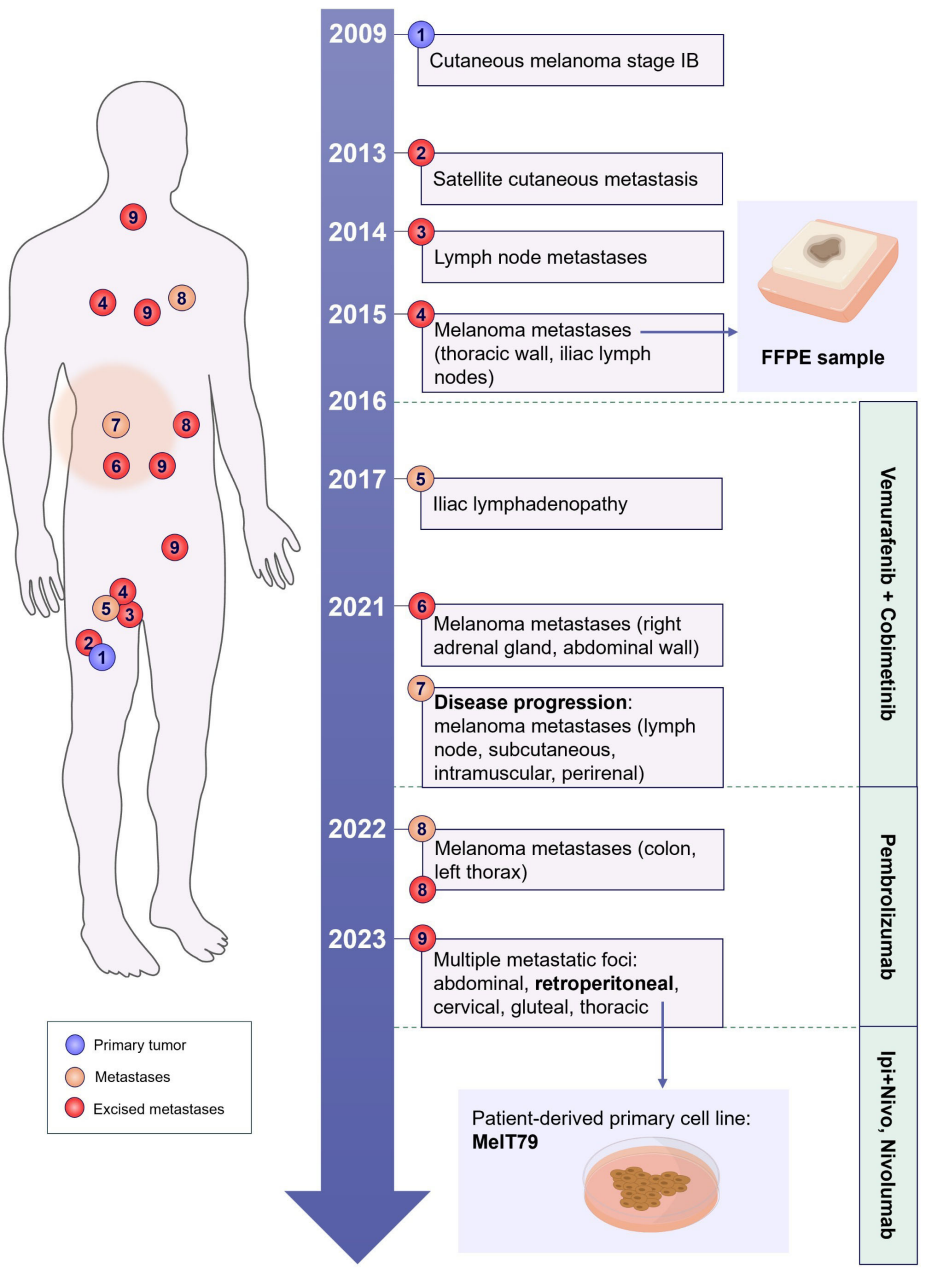
Schematic representation of the patients’ clinical history. The patient was diagnosed with stage IB cutaneous melanoma in 2009, located in the anterior right thigh. The timeline illustrates the development of metastatic lesions over the years, marking their approximate anatomical positions on the body schematic (merely illustrative). The treatments administered across different time periods are outlined on the right. A patient-derived primary cell line (MelT79) was established from one of the excised metastases in 2023, and an FFPE sample from a 2015 metastasis was also analyzed in this study, indicated by the small blue arrows. FFPE, formalin-fixed paraffin-embedded; Ipi+Nivo, ipilimumab+nivolumab.

The patient’s history is summarized in **Figure 1**. This study was approved by the Instituto Português de Oncologia de Lisboa Francisco Gentil (IPOLFG) Ethics Board Committee (UIC/1310) and written informed consent was obtained from the patient.

### 2.2 Cell culture

During metastases excision in 2023, a surgical fragment from a retroperitoneal metastasis was taken for cell culture. The tumor fragment was mechanically digested under sterile conditions with a scalpel (~1 mm diameter pieces). Subsequently, fragments were seeded in 1 well of a 24-well tissue culture plate in 500 μL McCoy’s 5A Medium (American Type Culture Collection, ATCC, 30-2007) supplemented with 10% Fetal Bovine Serum (FBS) (Corning 35-079-CV) and 1% Pen Strep (P/S) (Gibco 15140-122, Thermo Fisher Scientific). The petri dish where the digestion took place was also washed with 1 mL McCoy’s medium and placed in 2 additional wells (500 μL per well). Media was replenished every other day until wells reached confluency, one week later. Cells were detached with TrypLE™ Express Enzyme (Gibco 12605-028, Thermo Fisher Scientific) for approximately 5 min and subsequently sub-cultured. This primary cell culture was designated MelT79.

Commercially available human malignant CM cell lines A375 (ATCC CRL-1619, BRAF V600E, non-pigmented) and WM115 (Rockland WM115-01-0001, BRAF V600D, non-pigmented) were cultured in Dulbecco’s modified Eagle’s medium (DMEM) high glucose (Gibco 41965-039, Thermo Fisher Scientific), supplemented with 10% FBS and 1% P/S. G-361 cells (ATCC CRL-1424, BRAF V600E, lightly pigmented) were cultured in McCoy’s 5A Medium supplemented with 10% FBS and 1% P/S. MNT-1 cells (ATCC CRL-3450, BRAF V600E, highly pigmented) were cultured in DMEM high glucose with pyruvate (Gibco 21969-035, Thermo Fisher Scientific) supplemented with 10% FBS, 1% P/S, 1% L-glutamine (Gibco 25030-081, Thermo Fisher Scientific) and 1X MEM non-essential amino acids (Gibco 11140-050, Thermo Fisher Scientific). Cells were detached with 0.05% Trypsin 0.53 mM EDTA (Corning 25-052-CI) for approximately 5 min and split to new culture vessels according to the experimental standard procedures.

All cell cultures were maintained in a humidified incubator at 37°C, 5% CO_2_, and were regularly checked for mycoplasma contamination using Universal Mycoplasma Detection Kit (ATCC 30-1012K).

### 2.3 Cytogenetic analysis

Metaphases of the MelT79 cell line were obtained at passage 15 and multicolor fluorescence *in situ* hybridization (M-FISH) analysis was performed with 24XCyte Human Multicolor FISH Probe (MetaSystems) according to the manufacturer’s protocol. Fluorochromes were sequentially captured in a Zeiss Imager Z1 microscope linked to the M-FISH CytoVision software (version 7.4, Leica Biosystems). Karyotypes were described according to the International System for Human Cytogenomic Nomenclature (ISCN) 2020 (16).

High resolution-comparative genomic hybridization (HR-CGH) analysis of MelT79 tumor cells was performed as previously described (17).

### 2.4 Mutational analysis

DNA was extracted from MelT79 cells using the NZY Tissue gDNA Isolation Kit (NZYTech MB13502) according to the manufacturer’s instructions. DNA was also isolated from a 2015 formalin-fixed paraffin-embedded (FFPE) metastasis (**Figure 1**), using the Maxwell^®^ RSC FFPE DNA kit (Promega) in the Maxwell^®^ RSC platform (Promega) according to the manufacturer’s instructions. After quantification using the Qubit^®^ 4 fluorimeter (Life Technologies), 50 ng of DNA were used to prepare the next-generation sequencing (NGS) library on the Magnis NGS Prep System (Agilent Technologies), following the manufacturer’s protocol. A custom multigene panel using oligonucleotide probes (SureSelect XT HS2 DNA Target Enrichment, Agilent Technologies), routinely used in our laboratory, was employed to target cancer-related genes, including those frequently altered in melanoma, such as *BRAF*, *NRAS*, *CDKN2A*, *KIT*, *NF1*, *PTEN*, and *TERT* (complete list of genes on request). NGS was performed on a MiSeq platform (Illumina) with 75bp paired-end reads with average target coverage of 100x for the FFPE sample and 560x for the MelT79 cell line. Alignment and annotation were performed on the SeqOne platform using the SomaVar v2.4 workset (SeqOne Genomics) to the human reference genome GRCh37. The criteria for selecting potentially relevant gene variants included: base coverage > 20x, variant allele frequency (VAF) > 5% and clinical evidence of actionable, prognostic and diagnostic variants, provided by the Cancer Knowledge Base (CKD) database.

*BRAF* mutational status was validated by Sanger Sequencing, as previously described (18), using forward primer 5’ AAA CTC TTC ATA ATG CTT GCT CTG 3’ and reverse primer 5’ GGC CAA AAA TTT AAT CAG TGG A 3’.

### 2.5 Gene expression analysis

Total RNA was extracted and purified from all cell lines using NZY Total RNA Isolation kit (NZYTech MB13402), according to the manufacturer’s instructions, and quantified using Nanodrop 2000 (Thermo Fisher Scientific). Then, gene expression was assessed by reverse transcription quantitative PCR (RT-qPCR), as previously described (19). Primers used are presented in **Supplementary Table 1**, and *TBP* and *HPRT1* were selected as housekeeping genes.

### 2.6 Immunofluorescence analysis

Cells were seeded in duplicate in 24-well tissue culture plates containing glass coverslips pre-coated with 0.2% gelatin (Sigma-Aldrich G-1890), at a density of 1 × 10^5^ cells/well, and cultured for 48 h. At the endpoint, cells were washed three times with phosphate buffered saline 1x (PBS) and fixed with 2% paraformaldehyde for 20 mins at room temperature (RT). Permeabilization was performed with 0.1% Triton X-100 for 5 mins at RT, followed by three additional PBS 1x washes. Cells were then incubated for 10 mins with 50 mM NH_4_Cl at 4°C to quench autofluorescence. Blocking and further permeabilization were performed for 40 mins at RT using PermBlock solution (PBS 0.5% bovine serum albumin [BSA] 0.1% saponin). Cells were incubated with primary antibodies rabbit anti-MITF (1:200, overnight, at 4°C; Assay Biotech B0512) and mouse anti-TRP1 (1:200, 2h, at RT; Abcam ab3312), followed by three 5 mins washes to remove unbound antibodies. Secondary antibody incubation was performed using Alexa Fluor™ 488 goat anti-rabbit (1:1000, 2h, at RT; Invitrogen A11008) and Alexa Fluor™ 568 goat anti-mouse (1:500, 2h, at RT; Invitrogen A11004), followed by three additional 5 mins washes. Cells were then rinsed once with PBS 1x for 5 mins before coverslips were mounted onto glass slides with Vectashield^®^ Antifade Mounting Medium with DAPI (Vector Laboratories H-1200-10). All antibody dilutions and washes were performed using PermBlock solution. Images were acquired in a Zeiss Imager Z1 microscope linked to the CytoVision software (version 7.1, Leica Biosystems), and corrected total cell fluorescence (CTCF) quantification was performed using ImageJ software (version 1.53e). Intra-cell line heterogeneity was assessed by calculating, for each imaged field, the ratio of the CTCF range (maximum minus minimum value within that image) divided by the mean CTCF. This calculation was only applied to cell lines with detectable protein expression.

### 2.7 Doubling time calculation

Cells were seeded in duplicate in 6-well tissue culture plates at a density of 2 × 10^5^ cells/well. At each timepoint (24, 48 h), cells were detached as previously described in section 2.2. to create a cell suspension, which was mixed with Trypan blue solution 0.4% (Canvax Biotech CC007) in a 1:1 ratio. Viable cells were counted with a Neubauer improved cell counting chamber under the microscope. The cell doubling time was calculated using the following formula:


$$\text{Doubling}\;\text{Time}=\frac{\text{duration}\times \text{ln}\left(2\right)}{\text{ln}\left(\text{final}\;\text{cell}\;\text{number}\right)-\text{ln}\left(\text{initial}\;\text{cell}\;\text{number}\right)}$$

### 2.8 BrdU incorporation

Cell proliferation was evaluated using a colorimetric BrdU ELISA kit (Roche 11647229001), following the manufacturer’s instructions. Briefly, cells were seeded in triplicate in 96-well tissue culture plates at a density of 1 × 10^4^ cells/well, and cultured for 24 h. Two hours before the endpoint, cells were labeled with BrdU. At the endpoint, cells were fixed and incubated with a peroxidase-conjugated anti-BrdU antibody for 90 mins. After washing, cells were incubated with substrate solution for 10 mins, and the reaction was subsequently stopped with 1M H_2_SO_4_. Absorbance was measured at 450 nm using the iMark™ Microplate Absorbance Reader (Bio-Rad). For each each cell line, background absorbance values (from wells incubated with anti-BrdU only) were subtracted from the corresponding test wells.

### 2.9 Half-maximal inhibitory concentration determination

Cells were seeded in triplicates in 24-well tissue culture plates at a density of 5 × 10^4^ cells/well and allowed to adhere for 24 h. Then, cells were exposed to vehicle (dimethyl sulfoxide, DMSO, 1%), or different concentrations (0.1, 1, 10, 50, 100 nM, 1 μM) of trametinib (Selleckchem GSK1120212) or dabrafenib (Selleckchem GSK2118436) for 72 h. At the endpoint, Cell Counting Kit-8 reagent (Dojindo CK04-20) was added to each well in a 1:10 dilution, followed by a 30-minute incubation period at 37°C with 5% CO_2_, in the dark. Absorbance values were measured at 450 nm in a 96-well plate, using the iMark™ Microplate Absorbance Reader (BioRad).

### 2.10 Statistical analysis

Assays were performed with at least three biological replicates. All statistical analyses and graphical representations were performed using GraphPad Prism version 8.4.3.

Z-score values were calculated for each cell line regarding the expression of each gene, considering the mean gene expression value of all cell lines and the corresponding standard deviation (SD).

Data for relative gene expression, CTCF fold change, CTCF range/mean, cell doubling time, and cell proliferation are presented as mean with SD. Differences between MelT79 and the other cell lines were determined by one-way analysis of variance (ANOVA), followed by Dunnett’s multiple comparisons test to compare individual means.

IC_50_ values were calculated using a non-linear regression model, based on a dose-response slope, and are presented as best-fit IC_50_ values with lower and upper 95% confidence limits. Viability percentages are presented as mean with SD. Differences in IC_50_ values between MelT79 and commercially available cell lines were determined by extra sum-of-squares F test, with multiple comparison correction.

## 3 Results

### 3.1 Establishment of a cutaneous melanoma primary cell line

**Figure 2:**
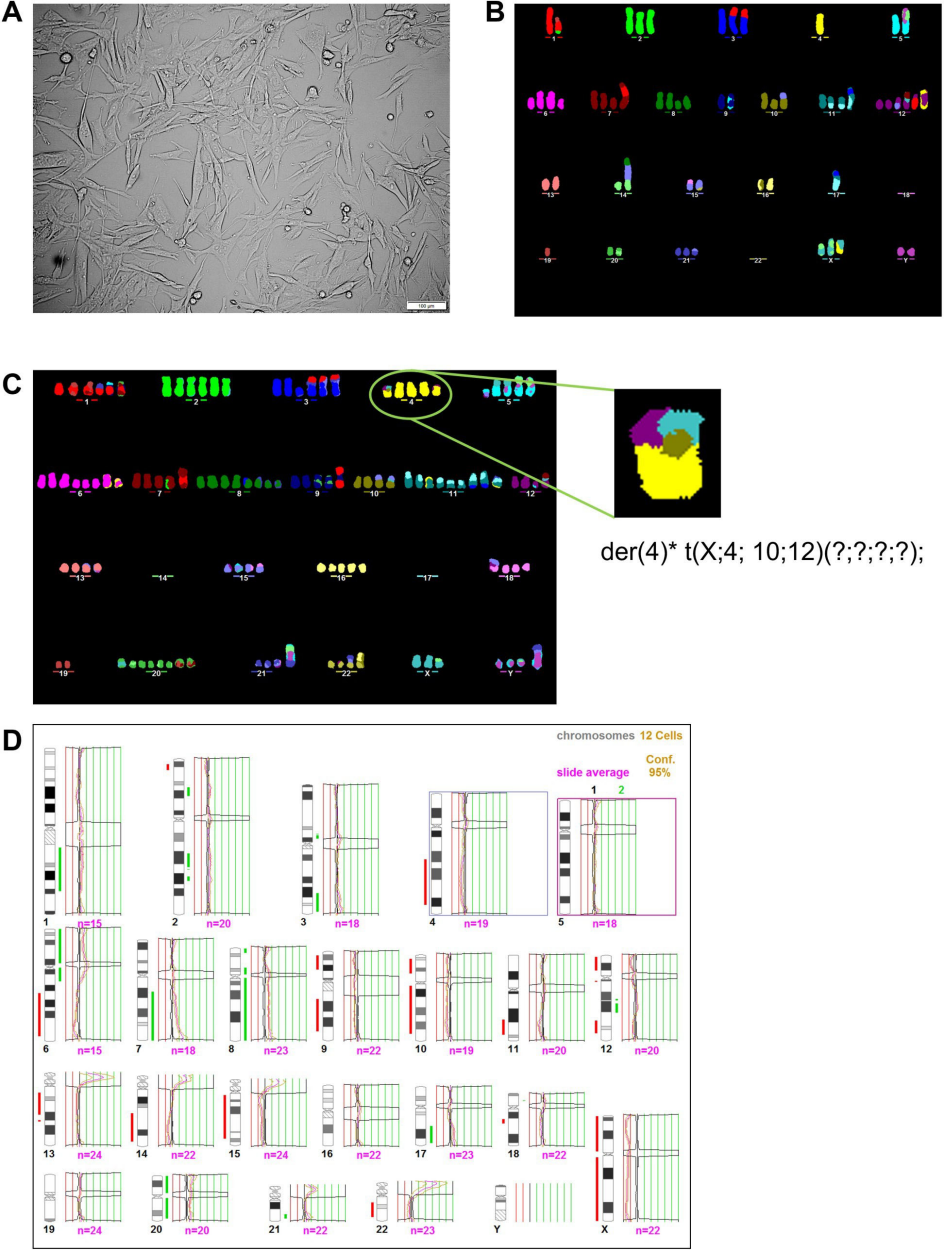
Patient-derived cutaneous melanoma cell line is highly heterogeneous, exhibiting a very unbalanced genome. **(A)** Light microscopy view from MelT79 patient-derived CM cell line, in passage 15 (scale bar, 100 μm). **(B)** M-FISH image of one MelT79 cell reveals a near-diploid karyotype. **(C)** M-FISH image of another MelT79 cell reveals a near-pentaploid karyotype, with a high number of inter-chromatid chromosomal aberrations, such as the one affecting chromosome 4. **(D)** HR-CGH analysis of MelT79 cells reveals multiple gains and losses of genetic material. A total of 12 metaphases were analyzed. The total number of chromosomes evaluated by CGH in the pool of metaphases is represented by “n=“ under each chromosome. Chromosomal imbalances were determined with a 95% statistical confidence level. Chromosomal gains are represented by the green segments, while losses are represented by the red segments, next to each chromosome.

Our group has made several attempts to establish primary cell cultures from CM surgical fragments, which have been faced with 3 main challenges, often leading to cell culture loss: (i) low melanoma cell viability, with no cell adhesion; (ii) cell senescence; (iii) fibroblast overgrowth. Out of 34 attempts and optimizations, this was the first successful primary cell line established from a metastatic CM surgical fragment in our group (**Figure 2A**).

Regarding the first challenge, studies have shown that enzymatic digestion processes can significantly impact cell viability (20, 21). Therefore, similarly to Ścieżyńska et al. (22), we processed tissue fragments through mechanical digestion only, to ensure maximum CM cell viability.

Najem et al. (23) have described that primary cell cultures growing in media with low tyrosine levels could be propagated through multiple passages, while maintaining a clinically relevant and highly differentiated melanoma state. The authors noted that some primary cultures grown in high-tyrosine media became rapidly senescent, leading to culture loss. Therefore, to prevent cell culture loss, we used McCoy’s 5A medium, which has a lower tyrosine concentration (26.1 mg/L) compared to DMEM (104 mg/L), the media we grow most CM cell lines in. We also hypothesized that this approach would help the culture retain a phenotype closer to the original tumor.

With this specific tumor fragment, we encountered no issue with fibroblast growth. Raaijmakers et al. (14) have already demonstrated that with tissues with a low fibroblast content, a simple protocol can be used to obtain primary cultures that adequately mirror the original tumor. Guo & Jahoda (24) demonstrated that fibroblasts begin migrating from tissue fragments 7 days after culture initiation. We removed most tissue fragments from our culture 2 days after seeding, during media change, retaining only the melanoma cells that had already adhered to the culture vessel surface.

Overall, there is a lack of studies that empirically demonstrate the optimal method to establish primary cell cultures from CM patients, with methodologies used varying widely (14, 22, 23, 25–28). Most studies involving primary CM cell cultures do not discuss the methodology in detail, nor do they compare failed attempts with the successful ones to clearly define key steps in the protocol. However, such studies are extremely challenging due to the high heterogeneity in tissue type, fragment size, tumor purity, and patient clinicopathological characteristics. As such, a trial-and-error approach is often employed for each case.

### 3.2 MelT79 cells exhibit a heterogeneous karyotype with multiple chromosomal gains and losses

M-FISH analysis revealed that the MelT79 cell line presents a complex karyotype with metaphase chromosomal number varying between near-diploid (n = 8) (**Figure 2B**, **Supplementary Table 2**) to near-pentaploid (n = 6) (**Figure 2C**, **Supplementary Table 2**). MelT79 cells are characterized by a high number of non-clustered structural chromosomal abnormalities such as deletions, inversions, and inter-chromosomal translocations. We also observed a high number of inter-chromatid chromosomal aberrations, such as the one depicted in **Figure 2C**. We have described this aberration affecting chromosome 4 as a der (4) t(X; 4;10;14) (?;?;?;?). These findings are in accordance with the observations made by Liu et al. (29) in therapy-resistant melanomas, who evidenced that these non-clustered structural aberrations were due to genetic defects in homologous recombination DNA repair and non-homologous end-joining (NHEJ) or alternative NHEJ recombination mechanisms.

Additionally, evaluation of MelT79 cells by HR-CGH revealed a highly unbalanced genome with multiple gains and losses (**Figure 2D**, **Supplementary Table 3**). Interestingly, and as reported by Liu et al. (29), we found that the three most significantly deleted chromosomal regions in their tumors – 9p21 (51%), 15q14 (45%) and 15q21.1 (45%) – are also deleted in MelT79 cells.

### 3.3 MelT79 cells harbor possibly targetable RET S649L mutation

To further characterize the alterations present in MelT79 cells, we have screened for gene mutations by NGS, using a multigene panel. Two gene mutations – BRAF V600E and RET S649L (**Table 1**) – have been detected, each with a VAF of 100%, confirming the absence of fibroblast contamination in the culture. BRAF V600E had already been previously identified in a 2015 cutaneous metastasis, which led to the implementation of targeted therapy with Vemurafenib and Cobimetinib. This mutation is the most common and clinically significant targetable mutation in CM (1).

**Table 1:** Relevant mutations detected by NGS in the MelT79 cell line and in an earlier, treatment-naïve patient metastasis from 2015. Mutations shared between the MelT79 cell line and the 2015 metastasis are highlighted in bold.

Sample	Gene	Chr	Exon	VAF	dbSNP ID	Type	HGVS nomenclature
MelT79 cell line (2023)	*BRAF*	7	15	100%	rs113488022	Missense	**NM_004333.6:c.1799T>A p.(Val600Glu)**
	*RET*	10	11	100%	rs148935214	Missense	**NM_020975.6:c.1946C>T p.(Ser649Leu)**
FFPE CM metastasis (2015)	*SDHC*	1	5	11.4%	rs760678574	Missense	NM_003001.5:c.295T>A p.(Tyr99Asn)
			5	10.4%	rs896411432	Missense	NM_003001.5:c.307G>C p.(Val103Leu)
	*PIK3CA*	3	12	10%	rs2108410770	Nonsense	NM_006218.4:c.1789C>T p.(Gln597Ter)
	*SDHA*	5	10	13.2%	rs143798161	Missense	NM_004168.4:c.1414G>A p.(Glu472Lys)
	*BRAF*	7	15	51.7%	rs113488022	Missense	**NM_004333.6:c.1799T>A p.(Val600Glu)**
	*RET*	10	7	10%	rs2132765897	Missense	NM_020630.6:c.1274T>A p.(Val425Asp)
			11	66%	rs148935214	Missense	**NM_020630.6:c.1946C>T p.(Ser649Leu)**
	*PTEN*	10	_	10%	rs587782455	Splice acceptor	NM_000314.8:c.802-2A>G p.?
	*POLE*	12	13	10.3%	rs151273553	Missense	NM_006231.4:c.1337G>A p.(Arg446Gln)
			33	10%	rs2042215726	Missense	NM_006231.4:c.4202C>T p.(Ser1401Leu)
			42	13.6%	rs778190944	Missense	NM_006231.4:c.5795G>A p.(Arg1932His)
	*ERBB2*	17	6	13.3%	rs760895559	Missense	NM_004448.4:c.676C>T p.(Arg226Cys)

VAF, variant allele frequency; dbSNP, Single-Nucleotide Polymorphism Database; HGVS, Human Genome Variation Society; FFPE, formalin-fixed paraffin-embedded.

To determine whether RET S649L emerged as a mechanism of targeted or immunotherapy resistance, or as an early event in disease progression, we performed NGS on an FFPE sample of the 2015 treatment-naïve metastasis, as no primary tumor sample was available. Given the degraded DNA quality in older FFPE samples, initial analysis identified an extensive list of 279 variants, most of which were likely false positives caused by DNA fragmentation. Therefore, we focused our analysis on the variants with VAF > 10% (**Table 1**), as previously discussed for low-quality FFPE samples (30). We confirmed the presence of both BRAF V600E and RET S649L in this earlier metastasis, indicating that RET S649L was an early event in tumor progression. Its persistence across different therapeutic approaches suggests that it may confer a selective advantage, making it a potential therapeutic target in this case, with a high likelihood of this mutation being present in most metastatic lesions of this patient.

Additional variants were identified in the 2015 FFPE metastatic sample, in genes *SDHA*, *SDHC*, *PIK3CA*, *PTEN*, *ERBB2*, *POLE*, and *RET*, but were absent in MelT79, which may reflect clonal evolution, highlighting CM’s dynamic genetic landscape. These genes are related with the PI3K/Akt and MAPK pathways (*PIK3CA*, *PTEN*, *ERBB2*) (1, 31), succinase dehydrogenase subunits (*SDHA*, *SDHC*) (32), and the DNA polymerase (*POLE*) (33). However, these variants are present at significantly lower VAF compared to RET S649L and BRAF V600E, which persisted throughout disease progression. This suggests that these variants are likely passenger mutations, artifacts, or subclonal events that were ultimately lost during progression or culture establishment. In contrast, the persistence of RET S649L and BRAF V600E underscores their potential roles as key drivers in this patient’s tumor evolution.

### 3.4 MelT79 cells display a differentiated phenotype with reduced proliferation

CM’s heterogeneity and plasticity enable tumor cells to adapt to microenvironmental changes and trigger metastasis and therapy resistance (34). Unlike other cancers, CM cells do not undergo epithelial-mesenchymal transition, since melanocytes are not epithelial cells, but instead use phenotype switching to transition between different states – from hyper-differentiated and slow-proliferating, to rapidly proliferating, or even slow-proliferating but highly invasive states (34–37).

**Figure 3:**
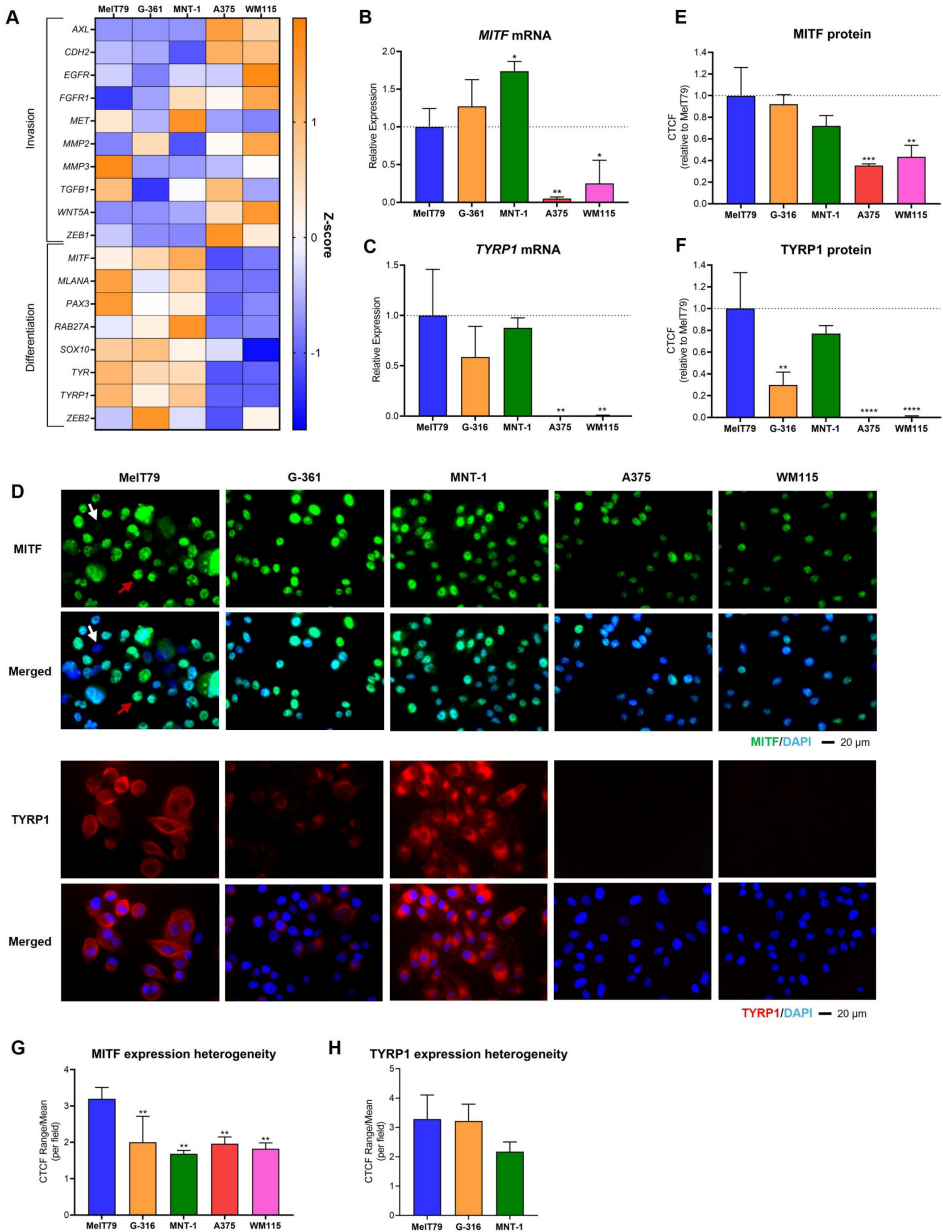
MelT79 cells share molecular expression similarities with G-361 and MNT-1 cell lines. **(A)** Heatmap showing the relative gene expression of multiple genes related with cutaneous melanoma (CM) invasion and differentiation across different CM cell lines, assessed by RT-qPCR. *TBP* and *HPRT1* were used as the endogenous control. Orange indicates an increase in gene expression compared to other cell lines, while blue indicates downregulation. **(B)** RT-qPCR analysis of *MITF* mRNA expression across CM cell lines. **(C)** RT-qPCR analysis of *TYRP1* mRNA expression across CM cell lines. **(D)** Representative immunofluorescence images showing MITF (green) and TYRP1 (red) protein expression. Nuclei are stained with DAPI (blue). White arrow highlights a MelT79 cell with low MITF expression; red arrow indicates a MelT79 cell with high MITF expression. **(E)** Corrected total cell fluorescence (CTCF) quantification of MITF levels across CM cell lines, using ImageJ. **(F)** CTCF quantification of TYRP1 levels across CM cell lines, using ImageJ. **(G)** Ratio of the CTCF range (maximum minus minimum value within a single image) divided by the mean CTCF, per imaged field, representing intra-cell line heterogeneity of MITF expression. **(H)** CTCF range-to-mean ratio for TYRP1 expression heterogeneity (limited to TYRP1-expressing cell lines). Data are presented as mean, standard deviation. One-way ANOVA followed by Dunnett’s multiple comparisons test was used to compare expression between MelT79 and the other cell lines. *p < 0.05, ** *p* < 0.01, ***p < 0.001, ****p < 0.0001. Experiments were performed with at least three biological replicates.

Given the extensive and complex clinical history of the patient from whom MelT79 cells were derived, our goal was to better understand the phenotypic characteristics that might contribute to the persistence of these cells despite different therapeutic approaches. Thus, we analyzed the expression of several genes involved in CM differentiation and invasion processes and compared it to commercially available CM cell lines with different pigmentation levels and growth patterns (**Figure 3A**).

MelT79 cells share similarities with G-361 and MNT-1 cell lines, upregulating melanin production genes linked to a differentiated phenotype (*MLANA*, *SOX10*, *PAX3*, *TYR*) (38, 39), while downregulating genes associated with invasion (*AXL*, *EGFR*, *CDH2*, *FGFR1*, *MMP2, WNT5A, ZEB1*) (23, 36). In contrast, A375 and WM115 cells exhibit a more invasive gene expression profile. However, despite the similar profiles, MelT79 differs from G-361 and MNT-1 in some respects: it expresses intermediate *MITF* levels, significantly lower than MNT-1 but higher than A375 and WM115 (**Figure 3B**); it displays elevated expression of the invasion-associated gene *MMP3*; and reduced expression of differentiation markers *RAB27A* and *ZEB2* (40–42). Despite these differences, *TYRP1*, a direct transcriptional target of *MITF*, closely linked to pigmentation (39), remains strongly expressed in MelT79 cells, consistent with the more differentiated profile shared with MNT-1 and G-361, and contrasting with A375 and WM115, which do not express it (**Figure 3C**).

To further characterize the differentiation status of MelT79 cells, we assessed MITF and TYRP1 protein levels by immunofluorescence. MITF protein expression in MelT79 is comparable to that in MNT-1 and G-361 cells, and markedly higher than in A375 and WM115, despite intermediate mRNA expression levels (**Figures 3D, E**). Notably, MITF expression varied widely from cell to cell within the same captured field, with some cells exhibiting high MITF levels, while others barely expressed it (**Figure 3D**). This heterogeneity was significantly greater in MelT79 when compared to the other cell lines (**Figure 3G**). TYRP1 protein levels were also elevated in MelT79, similar to MNT-1 and higher than G-361, consistent with mRNA trends (**Figure 3F**). However, TYRP1 expression showed no significant heterogeneity among MelT79 cells, indicating a more uniform protein expression (**Figure 3H**).

These expression patterns are closely tied to pigmentation status (39, 43) – pigmented cell lines G-361 and MNT-1 upregulate differentiation genes, while non-pigmented A375 and WM115 cell lines downregulate them. Despite the upregulation of most differentiation genes analyzed, with confirmed higher MITF and TYRP1 protein expression, MelT79 cells appear surprisingly non-pigmented (**Supplementary Figure 1**).

**Figure 4:**
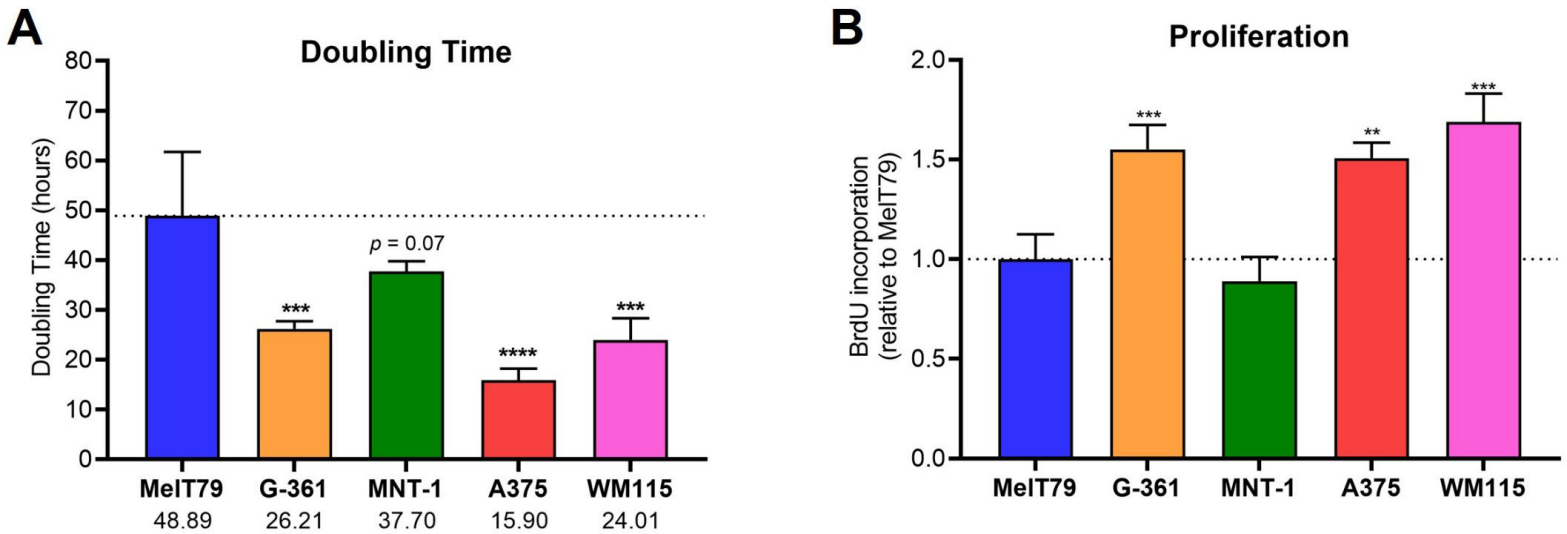
MelT79 cells show reduced proliferation. **(A)** Doubling times of selected cutaneous melanoma (CM) cell lines. Viable cells were counted with a Neubauer improved cell counting chamber at 24 and 48 h to calculate doubling times. **(B)** Relative proliferation of CM cell lines, measured by BrdU incorporation at 24 h using a colorimetric ELISA assay and normalized to MelT79. Data are presented as mean, standard deviation. One-way ANOVA followed by Dunnett’s multiple comparisons test was used to compare MelT79 with other cell lines. **p < 0.01, ***p < 0.001, ****p < 0.0001. Experiments were performed with at least three biological replicates.

Regarding doubling time, MelT79 exhibits significantly slower growth (49 h) compared to A375, WM115, and G-361. While their doubling time is closer to that of MNT-1 cells, there is still a trend indicating that MelT79 proliferates at a slower pace than MNT-1 cells (**Figure 4A**). Doubling times from patient-derived CM cell lines can vary widely (reports range from 25 to 104 h), depending on tissue origin, molecular alterations, and cell culture conditions (22, 28). These findings are supported by BrdU incorporation assay, which confirms that MelT79 cells proliferate significantly more slowly than G-361, WM115, and A375, and at a rate comparable to MNT-1 (**Figure 4B**).

Overall, the fact that MelT79 exhibits a differentiated gene expression profile, variable *MITF* expression when compared to other cell lines, lack of pigmentation, and reduced proliferation, further highlights this cell line’s heterogeneity.

### 3.5 MelT79 cells exhibit moderate sensitivity to BRAFi and MEKi, intermediate to invasive and differentiated cell lines

**Figure 5:**
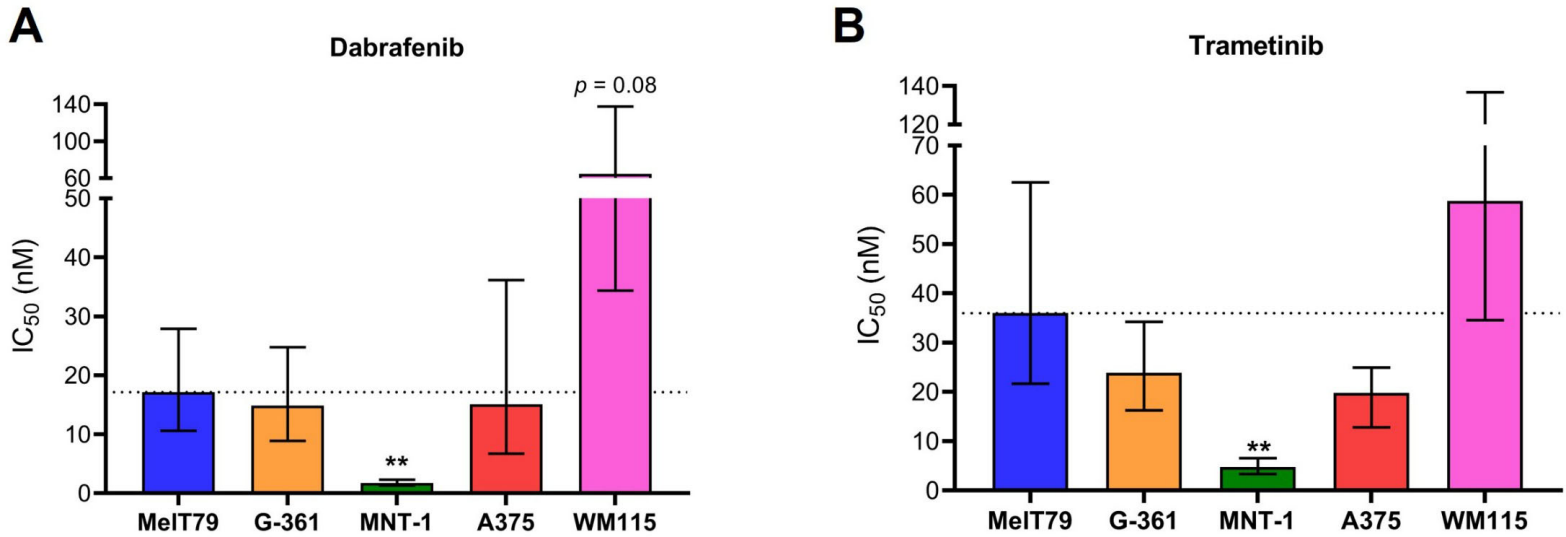
MelT79 cells are more resistant to BRAF and MEK inhibitors than highly differentiated MNT-1 cells. **(A)** Dabrafenib and **(B)** trametinib IC_50_ values of multiple cutaneous melanoma cell lines. Cells were exposed to different dabrafenib and trametinib concentrations for 72 h, and metabolic viability was assessed using Cell Counting Kit-8. IC_50_ values were calculated using a non-linear regression model and are presented as best-fit values with lower and upper 95% confidence limits. Differences in IC_50_ values between MelT79 and each commercial cell line were determined by extra sum-of-squares F test, with multiple comparisons correction. **p < 0.001. Experiments were performed with at least three biological replicates.

In advanced CM, targeted therapy is a key therapeutic approach for patients with tumors harboring *BRAF* mutations. Currently, this involves combination of BRAFi/MEKi, namely dabrafenib/trametinib (7). To assess MelT79 sensitivity to these inhibitors, we calculated their IC_50_ values and compared them to various commercially available CM cell lines (**Figure 5**, **Supplementary Figure 2**).

Our results show that IC_50_ values for both dabrafenib and trametinib are significantly higher in MelT79 cells compared to MNT-1 cells, indicating greater resistance. However, we observed a trend for both dabrafenib and trametinib’s IC_50_ to be lower in MelT79 cells compared to WM115. Since the transition from a differentiated to an invasive state is associated with increased resistance to targeted therapies (44, 45), it is expected that MNT-1 cells are the most sensitive, while WM115 cells seem to be the most resistant.

Interestingly, although MelT79 cells primarily exhibit a differentiated gene expression profile, our previous results show they also possess some dedifferentiated traits. This aligns with their intermediate sensitivity to BRAFi and MEKi when compared to the other cell lines – more resistant than highly differentiated MNT-1, but less resistant than the highly invasive WM115.

## 4 Discussion

The establishment of the MelT79 cell line from a metastatic lesion of a CM patient provides a valuable resource for studying the mechanisms underlying CM progression, therapy resistance, and phenotypic plasticity. This case is particularly notable as the patient was initially diagnosed at an early, low-risk stage (IB), yet experienced continued disease progression over 14 years despite different therapeutic approaches, highlighting the aggressive potential of certain CM subtypes and the need for improved risk stratification, even in early-stage CM. In the context of stage I CM, there is no consensus on follow-up methodology (7). Despite the excellent prognosis, there is still an unmet need for biomarkers that can better identify patients at higher risk of relapse. Furthermore, there is a lack of studies regarding the influence of *BRAF* mutations on survival in early-stage CM, raising questions about whether early-stage patients might benefit from *BRAF* mutational testing to better guide follow-up strategies and personalize their care (46).

MelT79’s pronounced heterogeneity, marked by complex chromosomal abnormalities, reflects the inherent genomic instability of the tumor that originated it. The cell line exhibits a highly unbalanced genome, with critical chromosomal deletions in regions harboring tumor suppressor genes with potentially relevant involvement in tumorigenesis. Notably, deletions of interferon cluster genes and the *CDKN2A*/*B* locus at 9p21, *SPRED1* at 15q14, and *B2M* at 15q21.1 have been observed. *CDKN2A*/*B*, crucial in the regulation of the cell cycle, are frequently lost in cancer, particularly in CM. Their deletion often leads to centrosome overduplication, driving chromosomal instability, which may underlie the high frequency of inter-chromatid chromosomal aberrations observed in MelT79 cells (47). *SPRED1*, encoding a negative regulator of the MAPK pathway similar to NF1, is also commonly deleted in CM and has been linked to resistance to BRAFi (48). Additionally, the loss of *B2M*, which encodes the β2M component of the MHC Class I complex, impairs antigen presentation and has been associated with resistance to anti-PD-1 immunotherapy (49), thus potentially contributing to the patient’s poor response to pembrolizumab. Collectively, these deletions likely contribute to key mechanisms of cancer progression and therapy resistance, consistent with the patient’s observed ongoing disease progression.

Further underscoring MelT79’s heterogeneity, gene expression analysis reveals that MelT79 cells predominantly display a differentiated gene expression profile; however, they also exhibit intermediate *MITF* and *RAB27A* expression, alongside upregulation of invasion gene *MMP3*, when compared to other CM cell lines. Immunofluorescence analysis showed MITF protein levels more comparable to pigmented and differentiated cell lines, MNT-1 and G-361, but with pronounced cell-to-cell heterogeneity. MITF expression varied widely among MelT79 cells, ranging from high to barely detectable, a variability not observed in other cell lines. This suggests coexistence of distinct populations, possibly undergoing dynamic phenotypic transitions, in line with our observation of an intermediate transcriptional profile. In contrast, TYRP1, a direct MITF target involved in melanin synthesis (39), remains consistently high with less variability, implying its levels may be buffered by regulatory mechanisms despite fluctuations in MITF. MITF is known to regulate melanin synthesis (38), and its downregulation is usually linked to increased invasiveness (36, 37) and acquired therapy resistance (targeted and anti-PD1 therapies, which the patient had been submitted to prior to cell line establishment) (44, 50). Therefore, the presence of MelT79 subpopulations with reduced MITF expression might support the notion of a plastic cell line, shaped by microenvironmental pressures to adapt and survive. Interestingly, MelT79 cells are non-pigmented despite high MITF and TYRP1 expression. This complete lack of pigmentation could be attributed to low *RAB27A* levels, or other defects in melanosome trafficking, melanin synthesis, or melanin secretion. It is important to note, however, that pigmentation can be influenced by cell culture conditions (23, 25, 51). Together, these findings suggest that MelT79 cells retain aspects of a differentiated phenotype without acquiring full pigment-producing functionality, reinforcing their intermediate state and phenotypic plasticity.

Another notable feature of MelT79 cells is their slow proliferation. Mechanisms of CM phenotypic switching have been described, where cells transition from a proliferative to a slow-cycling, invasive state to drive metastasis, typically by downregulating differentiation genes (34, 35). However, this is not observed in MelT79 cells, which continue to express most differentiation-associated genes. Recent research suggests that these phenotypic states may not always be mutually exclusive, particularly during and after therapy exposure. Therapeutic pressure can promote the enrichment of slow-cycling cells in an intermediate phenotypic state that contributes to therapy resistance (36, 37, 52). Indeed, our findings indicate that MelT79 cells exhibit both differentiation and invasive traits – they may exist in this intermediate phenotypic state, or represent a highly heterogeneous population, as supported by the cell line’s complex karyotype, existing in varying differentiation states to drive disease progression and therapy resistance. This phenotypic plasticity likely enabled MelT79 cells to adapt to diverse microenvironments and evade therapies, contributing to disease progression, alongside the loss of *SPRED1* and *B2M* – both liked to poor response to targeted therapies (BRAFi/MEKi) and immunotherapy (anti-PD-1), respectively.

The identification of the rare RET S649L mutation in this cell line introduces a novel potential therapeutic target. *RET* is a proto-oncogene that encodes a receptor tyrosine kinase involved in the development of neural and genitourinary tissues. Activating *RET* mutations are commonly associated with familial medullary thyroid carcinoma (MTC) and multiple endocrine neoplasia syndrome type 2 (53). These mutations are also observed in sporadic MTC and lung cancers, making them important actionable targets (54). The RET S649L mutation, however, is rare, and has thus far only been reported in MTC patients (mostly familial, with one sporadic case) (55–58). *In vitro* studies suggest that RET S649L has a low aggressive potential in MTC – despite leading to an increase in kinase activity, cells harboring RET S649L showed a lower proliferation rate compared to those with RET C634R, a more prominent RET mutation (56). Furthermore, familial MTC patients with RET S649L typically carry a second, higher-risk RET mutation, supporting its classification as low-risk in the context of familial MTC (56, 57). Regarding therapy response, RET alterations have been linked to varying responses to immune-checkpoint inhibitors. In lung cancer, RET rearrangements have been associated with “cold” tumors and poor response to anti-PD1 therapies (59). Conversely, a multi-cancer study which included melanoma samples linked RET mutations to favorable immunotherapy outcomes, characterized by an increase in cytotoxic T cell infiltration and upregulation of immune checkpoints such as CTLA-4, PD-1 and PD-L1 (60) Thus, the precise role of RET S649L in sporadic CM, including its impact on tumor progression, immunotherapy response, and its viability as an actionable target in this setting, remains unknown, and further studies are needed. Nonetheless, the presence of RET S649L in two distinct metastatic lesions, coupled with its persistence throughout disease progression, suggests it may confer a selective advantage, positioning it as a promising candidate for further exploration as an actionable target.

It is important to note that this study has certain limitations. The absence of RNA sequencing data restricts the ability to thoroughly characterize gene expression changes driving disease progression. Additionally, no sample from the patient’s primary tumor was available for comparison, limiting the ability to trace the evolutionary trajectory of MelT79 cells from the beginning stage and hindering a comprehensive understanding of how these cells may have persisted and evolved from stage IB. These factors underscore the need for further studies to elucidate the mechanisms by which MelT79 cells persist and adapt during the disease course.

## 5 Conclusion

The establishment of the MelT79 cell line from a metastatic cutaneous melanoma lesion provides a valuable CM cell model that mirrors the complexity, phenotypic plasticity, and heterogeneity of advanced CM. This model offers new opportunities to study CM biology, test therapeutic strategies, and explore emerging targets such as *RET* mutations. The heterogeneous genetic and phenotypic traits of this novel cell line highlight the dynamic nature of CM and its ability to adapt to different therapeutic approaches. While our findings suggest that RET S649L may represent a novel therapeutic target, future research should focus on testing RET inhibitors in MelT79 cells to evaluate their therapeutic potential, as well as leveraging this cell line to uncover the potential role of *RET* mutations in immune-checkpoint blockade therapy response, which remains unclear. Exploring this patient-derived cell model further may lead to better personalized strategies for managing persistent CM and improving outcomes for patients with similar disease progression. This study also emphasizes the need for refined risk stratification in the management of CM patients.

## Data Availability

The sequencing data presented in this study are deposited in the European Nucleotide Archive (ENA) at EMBL-EBI under accession number PRJEB92110 (https://www.ebi.ac.uk/ena/browser/view/PRJEB92110).
