# Supplementary material for: A novel patient-derived cutaneous melanoma cell line reveals key features of metastatic melanoma
Source: Front Oncol. 2025 Jul 18;15:1531013. doi: 10.3389/fonc.2025.1531013 (PMC12313697; doi:10.3389/fonc.2025.1531013)
Supplement: Supplementary file 1 [file DataSheet1.docx]

Supplementary Material

**Supplementary Table 1**. Primer sequences used in RT-qPCR.

| **Gene** | **Forward primer (5’ → 3’)** | **Reverse primer (5’ → 3’)** |
| --- | --- | --- |
| *AXL* | GGATGAACAGGATGACTGGATAGTG | TGTCCCGTGTCGGAAAGC |
| *CDH2* | TCAGGCGTCTGTAGAGGCTT | ATGCACATCCTTCGATAAGACTG |
| *EGFR* | CCGTCGCTATCAAGGAATTAAG | CACGCTGGCCATCACGTAG |
| *FGFR1* | CGCCAGGACCCGAACAG | CAGTGAGCTCGATCCTCCTTT |
| *MET* | TGGGAATCTGCCTGCGAA | CCAGAGGAGCACGCCAAA |
| *MITF* | GCGCAAAAGAACTTGAAAAC | CGTGGATGGAATAAGGGAAA |
| *MLANA* | GATGCCAAGAGAAGATGCTCAC | GTCTCGCTGGCTCTTAAGGTGAA |
| *MMP2* | CTTCAAGGACCGGTTCATTTGG | GCCTCGTATACCGCATCAATC |
| *MMP3* | AGTCTTCCAATCCTACTGTTGCT | TCCCCGTCACCTCCAATCC |
| *PAX3* | GCCGCATCCTGAGAAGTAAA | CTTCATCTGATTGGGGTGCT |
| *RAB27A* | GAAGCCATAGCACTCGCAGAG | ATGACCATTTGATCGCACCA |
| *SOX10* | AGCTGGGCAAGGTCAAGAAG | TGGGCTGGTACTTGTAGTCC |
| *TGFB1* | CAAGGGCTACCATGCCAACT | AGGGCCAGGACCTTGCTG |
| *TYR* | TTGGCAGATTGTCTGTAGCC | AGGCATTGTGCATGCTGCTT |
| *TYRP1* | ATGGCAACACGCCACAATTTGAG | CCCGTTGCAAAATTCCAGTAAG |
| *WNT5A* | CCGCGAGCGGGAGCGCAT | GCCACATCAGCCAGGTTGTACACC |
| *ZEB1* | TTACACCTTTGCATACAGAACCC | TTTACGATTACACCCAGACTGC |
| *ZEB2* | AGGAGCAGGTAATCG | TGGGCACTCGTAAGG |
| *HPRT1* | TGACACTGGCAAAACAATGCA | GGTCCTTTTCACCAGCAAGCT |
| *TBP* | GAGCTGTGATGTGAAGTTTCC | TCTGGGTTTGATCATTCTGTAG |

**Supplementary Table 2.** Summary of the chromosomal alterations observed by M-FISH in the tumor metaphases depicted in Figures 2B and 2C. A total of 21 metaphases were observed – 8 were near‑diploid, 6 near-triploid, 1 near-pentaploid, and 6 near-pentaploid.

| **Figure** | **Karyotype** |
| --- | --- |
| 2B, near‑diploid metaphase | 58<46> der(X) ins (14;X)(?;?); + der(X)*t(X;14)(?;?); + der(X)* t(X;4)(?;?); +Y; der (1) t(1;2;19)(?;?;?); +2; der(3) t(1;3)(?;p?); + der(3) t(1;3)(?;p?); - 4; der(5) t(Y;5;14)(?;?;?); + del(6)(?q)x2; + del(7)(?), + der(7)ins(1;7)(?;?); + del (8)(p?); + del(8)(?); der(9) t(5;9)(?;?); del(10)(?); + der(10) t(10;15)(?;q?); der(11)t(11;17)(?;q?)x2; + der (11)t(3,11;17)( ?;?;?); del(12)(?p)x2; + der(12) t(12;15)(q?;q?); + der(12)t(X;7;12)(?;?;?); + der(12) t(1;12) (q?;p11~q11); + der (12)ins(4;12)(?;?); del (14)(q?); der(14) t(8;14;15) (?;?;?), der (15) t(15;18)(q?;?); der (15) t(15;22)(q?;q?); der(16)*t(10;16)(?;?); - 17; der(17) t(3,11;17)( ?;?;?); der (11)t(3,11;17)( ?;?;?); -18; -18; -19; der (20)ins (14;20)(?;?); + der(21) t(15;21)(q?,q?), -22; -22 |
| 2C, near‑pentaploid metaphase | 113 <115> -X; -X; der(X) t(X;14)(?;?); -Y; der (Y)t(Y;5;15)(?;?;?); der(Y) t(Y;X;5)(?;?;?); der(Y)t(Y;X;5;14) (?;?;?;?); der (Y)t(Y;5;15;21); del (1)(?); der(1) ins(1;19)(?;?); der(1)ins (1;7;19)(?;?;?); der (1)ins (1;5;9)*(?;?;?); der (1)ins (1;7; 9;20) (?;?;?;?); + der (1) t(1;21)(?:?); +2; der(3) del(3) t(3;21)(?;?); der(3)(1;3;21) (?;?;?); der(3)t(1;3;15)* (?;?;?) +der(3)t(1;3;15;21) (?;?;?;?); der(4)t(4;12)(?;?); der(4)t(X;4; 10;12)(?;?;?;?); der(5) t(X;5)(?;?); der(5)t(X;Y;5)(?;?;?); der(5) t(Y;5;14) (?;?;?); der(5)t(Y;5;14) (?;?;?); der(5)del(5)(?)t(5;12;15)(?;?;?); del(6)(p?15)x3; + der(6)t(4;6)(?;?); + der(6)t(4;6;18) (?;?;?); del(7)(p?15); der(7)del(7)t(2;7) (?;?); der(7)* t(1;7)((?;?); der(8)del(8) t(3;8) (?;?); + der(8) del(8) t(8,21)(?;?); + der(8)del(8)t(8,21)(?;?); + der(8)del(8)t(8;21)(?;?); der(9)ins(9;20) (?;?); der (9)ins(9;20) (?;?); iso der(9)t(1;9)(?;?); -10; der(10) t(10;15)(p?;q?); der(10) t(X;10;15)(?;?;?); der(11) t(11;17)(?;?) x3; der(11)t(11;17)(?;?); der(11) del(11)t(11;17)(p?;?); der(11)t(4;11)(?;?); + der(11)t(4;11;17;21)(?;?;?;?);+ der(11)t(3;11;17)(?;?;?) x2; -12; der(12)t(5;12)(?;?); der(12) t(X;7;12) (?;?;?); -13; der(13)t(13;21)(q?;q?); der(13)t(Y;13;15)(?;?;?); -14; -14; -14; -14; -14; -15; der(15)t(12;15)(?;q?); der(15)t(X;5;15)(?;?;q?); der(15) t(15;18)(q?;?); der(15) t(15;18)(q?;?); -17; -17; -17; -17; -17; -18; der(18)t(12;15;18)(?;?;?); -19; -19; -19; der(20)t(14;20)(?;?)x2; der(20) ins(14;20)(?;?); + der(20) ins(X;20)(?;?); +der(20) t(7;9;19;20) (?;?;?;?) x2; -21; der(21) t(15;21)(q?;q?); der(21)t(15;21)(?;?); der(21)(Y;5; 14;21)(?;?;?;?); der (21)t(12;15; 17; 18; 21)(?;?;?;?;?); -22; der(22) t(12;22)(?;?); der(22)t(12;22)(?;?); der(22) t(15;21;22)(?;?;?); tas(22)der(?16) add(22)* t(10;16) (?;?;?). |

| **Supplementary Table 3.** Summary of the HR-CGH imbalances observed in MelT79 cells. | |
| --- | --- |
| **Gains at chromosomal regions** | 1q21-q32; 2p21-p16; 2q22-q32; 3q26-q29; 6p25-q13; 7q21-q36; 8p23; 8p12-q24; 12q21-q23; 17q21.3-q25; 20p13-q13.3; e 21q22.3 |
| **Losses at chromosomal regions** | 2p24-p23.3; 4q22~24-q32~34; 6q21-q27; 9p23-p21; 9q13-q34.1; 10p14-p12; 10q11.2-q26.1; 11q23; 12p13-p21; 12q24.1-q24.3; 13q12-q14; 14q24-q32; 15q11.2-q24; 18q21.1; 22q11.2-q13. |

**
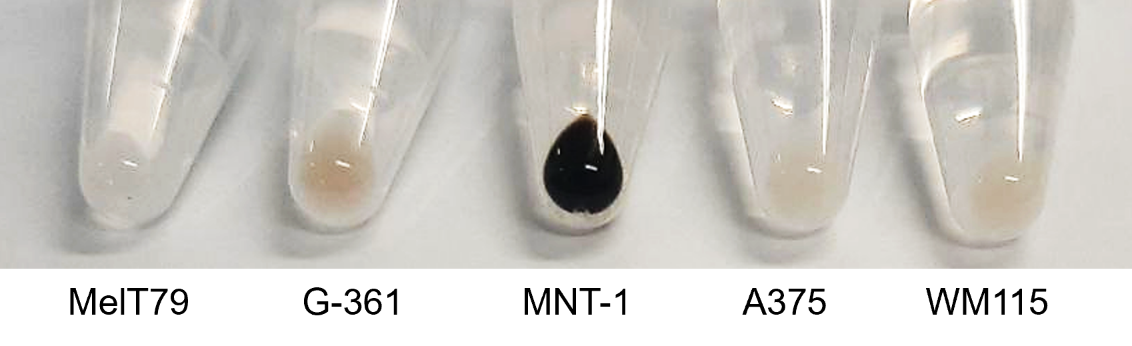
**

**Supplementary Figure 1**. Visible pigmentation comparison between the cell pellets of the patient‑derived primary cell line MelT79 and various CM cell lines.


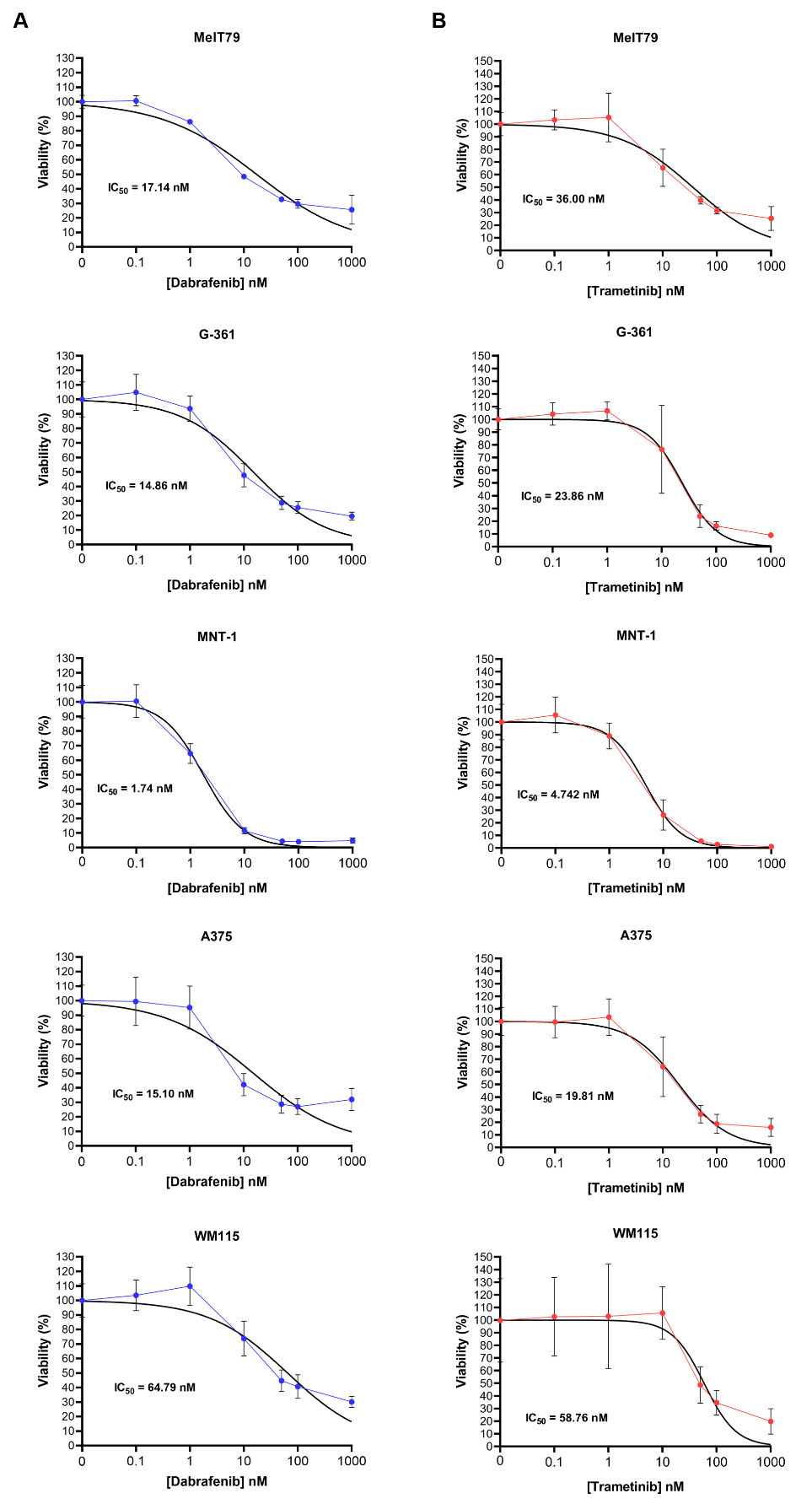


**Supplementary Figure 2**. (A) Dabrafenib and (B) trametinib dose-response curves and corresponding IC_50_ values for each cell line. Cells were exposed to different concentrations of dabrafenib and trametinib for 72 h and metabolic viability was assessed using Cell Counting Kit-8. Viability percentages are represented as mean and standard deviation. Experiments were performed with at least three biological replicates.
